# Playing the Whack-A-Mole Game: ERK5 Activation Emerges Among the Resistance Mechanisms to RAF-MEK1/2-ERK1/2- Targeted Therapy

**DOI:** 10.3389/fcell.2021.647311

**Published:** 2021-03-11

**Authors:** Alessandro Tubita, Ignazia Tusa, Elisabetta Rovida

**Affiliations:** Department of Experimental and Clinical Biomedical Sciences “Mario Serio”, University of Florence, Florence, Italy

**Keywords:** MAPK, ERK1/2/5, resistance mechanisms, combined therapy, targeted therapy, cancer

## Abstract

Molecularly tailored therapies have opened a new era, chronic myeloid leukemia being the ideal example, in the treatment of cancer. However, available therapeutic options are still unsatisfactory in many types of cancer, and often fail due to the occurrence of resistance mechanisms. With regard to small-molecule compounds targeting the components of the Mitogen-Activated Protein Kinase (MAPK) cascade RAF-MEK1/2-ERK1/2, these drugs may result ineffective as a consequence of the activation of compensatory pro-survival/proliferative signals, including receptor tyrosine kinases, PI3K, as well as other components of the MAPK family such as TPL2/COT. The MAPK ERK5 has been identified as a key signaling molecule in the biology of several types of cancer. In this review, we report pieces of evidence regarding the activation of the MEK5-ERK5 pathway as a resistance mechanism to RAF-MEK1/2-ERK1/2 inhibitors. We also highlight the known and possible mechanisms underlying the cross-talks between the ERK1/2 and the ERK5 pathways, the characterization of which is of great importance to maximize, in the future, the impact of RAF-MEK1/2-ERK1/2 targeting. Finally, we emphasize the need of developing additional therapeutically relevant MEK5-ERK5 inhibitors to be used for combined treatments, thus preventing the onset of resistance to cancer therapies relying on RAF-MEK1/2-ERK1/2 inhibitors.

## Introduction

The Mitogen-Activated Protein Kinase (MAPK) cascades are involved in a number of physiological processes and are activated by a large variety of stimuli. Conventional MAPKs include the four subfamilies of extracellular signal-regulated kinase 1 and 2 (ERK1/2), c-Jun N-terminal kinases 1–3, p38 α, β, γ, and δ, as well as ERK5. Atypical MAPKs have also been identified: ERK3, ERK4, ERK8 (also known as ERK7) and Nemo-like kinase (Cargnello and Roux, 2011).

Regarding the MAPK cascade culminating in ERK1/2 activation, a variety of mitogens activate receptor tyrosine kinases (RTKs) or G-protein-coupled receptors that, in turn, activate the small GTPase RAS proteins (K-RAS, H-RAS, or N-RAS) that are responsible for the recruitment of RAF kinases. Once activated, RAF-1 (also named c-RAF), ARAF or BRAF (Johnson and Lapadat, 2002; Roux and Blenis, 2004; Kolch, 2005) phosphorylate at S/T residues and thus activate MEK1 and 2, that in turn, phosphorylate T and Y residues at the TEY sequence of ERK1/2, leading to its activation. Activated ERK1/2 phosphorylates many substrates, including transcription factors and protein kinases (Yoon and Seger, 2006). Subsequently, immediate early genes controlling cell proliferation are rapidly induced (Lewis et al., 1998). The RAS-RAF-MEK1/2-ERK1/2 pathway regulates multiple critical cellular functions including survival, proliferation and differentiation (Cargnello and Roux, 2011). The alteration of this pathway has been frequently reported in several types of cancer as a result of abnormal activation of RTKs or gain-of-function mutations mainly in the *RAS* or *RAF* genes. Accordingly, RAF-MEK1/2-ERK1/2 inhibitors are among the therapeutic options for the treatment of many types of cancers (Sebolt-Leopold and Herrera, 2004; Roberts and Der, 2007; Montagut and Settleman, 2009; Holderfield et al., 2014; Roskoski, 2018). Unfortunately, several resistance events have been reported, so that combined treatments are often needed and actively sought after (Little et al., 2013; Samatar and Poulikakos, 2014; Liu et al., 2018; Lee et al., 2020).

ERK5, the most recently identified MAPK, is the effector kinase of a typical three-tiered MAPK cascade (Lee et al., 1995; Zhou et al., 1995; Nithianandarajah-Jones et al., 2012). In response to several stimuli, the S/T kinases MEKK2 or MEKK3 activate MEK5, a dual specificity protein kinase active on ERK5. Once activated, MEK5 phosphorylates two residues at the TEY sequence of ERK5 and induces ERK5 nuclear translocation. Besides sharing high homology with ERK2 in the kinase domain and exhibiting in the activation loop a TEY motif identical to that of ERK1/2/8, ERK5 has a long C-terminal tail that is unique among all MAPK. The C-terminal tail includes a nuclear localization sequence (NLS) important for ERK5 nuclear targeting, two proline-rich (PR) domains (PR1 and PR2), which are considered potential binding sites for Src-homology 3 (SH3)-domain-containing proteins, a nuclear export sequence (NES) and a myocyte enhancer factor 2 (MEF2)-interacting region (Yan et al., 2001). The C-terminus of ERK5 also possesses a transcriptional activation domain (TAD) (Kasler et al., 2000) that undergoes autophosphorylation, thereby enabling ERK5 to directly regulate gene transcription (Morimoto et al., 2007). Known ERK5 substrates include the transcription factors Sap-1a, c-Fos, c-MYC, and MEF2 family members (A, C and D), as well as kinases such as the ribosomal s6 kinase and the serum/glucocorticoid-regulated kinase (Wang and Tournier, 2006; Nithianandarajah-Jones et al., 2012; Hoang et al., 2017). Despite mediating proliferation and differentiation signals similarly to ERK1/2, ERK5 emerged since its very discovery to have distinct roles with respect to ERK1/2, and to mediate signals which cannot be compensated for by other MAPKs (Cavanaugh et al., 2001; Nishimoto and Nishida, 2006). Accordingly, ERK5 null mice die early in their development (E9.5-10.5) because of severe defects in vasculature and cardiac development, pointing to a critical role of ERK5 in controlling angiogenesis, at least in mice (Hayashi and Lee, 2004). In normal cells, the MEK5-ERK5 pathway plays a central role in supporting cell survival, proliferation, differentiation, and motility, as well as in repressing apoptosis. Along this line, it is not surprising that there is increasing evidence regarding the involvement of this pathway in tumor development and progression (Stecca and Rovida, 2019). Based on that, targeting the MEK5-ERK5 pathway has clearly emerged among the possible strategies to reduce cancer growth (Simões et al., 2016; Hoang et al., 2017).

In this paper, we describe the accumulating lines of evidence pointing to ERK5 activation as a compensatory mechanism occurring upon RAF-MEK1/2-ERK1/2 inhibition, and determining *de facto* the resistance to therapeutic strategies based on this inhibition. ERK5 targeting should therefore be exploited to become part of new combination treatments capable of enhanced effectiveness against several types of cancer.

## Evidence for ERK5 Activation as a Resistance Mechanism in RAS-Driven Cancers

Because effective RAS-directed therapies are still lacking, targeting RAS-downstream signals such as MEK1/2 and/or ERK1/2 using small-molecule compounds is among the strategies used in RAS-driven cancer. However, MAPK inhibitors (MAPKi) are not very effective when used as single agents, due to intrinsic and/or acquired resistance toward ERK1/2i and/or MEK1/2i (Little et al., 2013; Samatar and Poulikakos, 2014; Dummer et al., 2017). In this respect, a number of papers have described the relevant role of MEK5-ERK5 pathway in the lack of effectiveness of MAPKi in RAS-driven cancer.

The first report shedding light on this important issue showed that the activation of the MEK5-ERK5 cascade conferred insensitivity to MEKi in intestinal epithelial cells (IEC) and in K-RAS-mutated colo-rectal carcinoma (CRC) cells (de Jong et al., 2016). ERK1/2 pathway appeared to be dispensable for IEC proliferation, and either ERK1/2 genetic deletion in primary IEC or treatment of human CRC cell lines with the MEK1/2 inhibitor PD0325901 led to compensatory activation of ERK5. The authors proposed a model in which, when the ERK1/2 module is intact, RAS-dependent signaling preferentially activates the RAF-MEK1/2-ERK1/2 cascade. In this context, ERK1/2-dependent negative feedback mechanisms stimulate dual specificity phosphatases (DUSPs) (Lake et al., 2016) that restrain the ERK5 pathway. On the other hand, upon MEK1/2 inhibition or genetic knockout of ERK1/2, this feedback is blocked, resulting in the upregulation of the RAS-RAF-MEK5-ERK5 module, which maintains IEC and CRC cell proliferation. Consistently, targeting both pathways caused a more effective suppression of cell proliferation in both murine intestinal organoids (genetic ERK1/2 inhibition plus ERK5 inhibitor XMD8-92) and human CRC cell lines (PD0325901 + XMD8-92) (de Jong et al., 2016).

Other evidences of the central role of MEK5-ERK5 in the resistance to MAPKi in RAS-driven cancers emerged in pancreatic ductal adenocarcinoma (PDAC), where K-RAS is mutated in 95% of cases (Waters and Der, 2018). Vaseva et al. (2018) found that the treatment of human PDAC cell lines with the ERK1/2i SCH772984 led to compensatory phosphorylation/activation of the MEK5-ERK5 cascade. This activation promoted MYC protein stability as a consequence of phosphorylation at S62 by ERK5. Additionally, ERK1/2 inhibition caused a delayed increase in the phosphorylation of EGFR, HER2 and SRC, so that combined SCH772984/EGFRi (Poziotinib, Erlotinib) or SCH772984/SRCi (Saracatinib) prevented ERK5 phosphorylation. Based on all above, the authors proposed a model where ERK1/2 inhibition induces a EGFR/HER2/SRC-dependent feedforward activation of MEK5-ERK5, that prevents MYC degradation. Finally, concurrent inhibition of ERK5 (XMD8-92) and ERK1/2 (SCH772984) synergistically suppressed the growth of patient-derived PDAC xenografts. These results are of particular interest, as both RAS and MYC are very difficult to target directly (Dang et al., 2017).

K-RAS is the most commonly mutated member of the RAS family in non-small cell lung cancer (NSCLC) (Suzuki et al., 1990). MAPKi have proven ineffective in the treatment of NSCLC as much as in the other types of RAS-driven cancers (Carter et al., 2016; Jänne et al., 2017). Along this line, Dompe et al. (2018) found that the treatment of K-RAS-mutated NSCLC cell lines with the MEK1/2i Cobimetinib, that results in delayed activation of ERK1/2, increased ERK5 phosphorylation. Interestingly, ERK5 inhibition (XMD17-109) attenuated the re-activation of ERK1/2 signaling occurring upon MEK1/2 inhibition, pointing to a prominent role of ERK5 in mediating ERK1/2 reactivation upon MEK1/2 targeting. Finally, the combination of Cobimetinib (MEK1/2i) with the genetic knockdown of *MAPK7*, the gene encoding for ERK5, resulted more effective than single treatments in reducing the growth of K-RAS-mutated NSCLC xenografts (Dompe et al., 2018).

Advanced stage cutaneous melanoma is a highly malignant tumor characterized by somatic mutations of a number of oncogenes involved in the RAS-RAF-MEK1/2-ERK1/2 pathway, including N-RAS or BRAF, that lead to uncontrolled proliferation. MAPK pathway-targeting regimens are a valuable treatment option for BRAF-mutated melanoma (Luke et al., 2017; Ugurel et al., 2017). Unfortunately, patients with N-RAS mutation (around 20% of cases, e.g., N-RAS-Q61K/L) (Schadendorf et al., 2015) do not benefit from such therapies, owing to the lack of targetable BRAF mutations and a high degree of intrinsic and acquired resistance to MEK1/2 inhibition (Dummer et al., 2017). In keeping with a possible involvement of ERK5 in MAPKi resistance in N-RAS-mutated melanomas, a recent report showed that the treatment with MEK1/2i (Trametinib, Binimetinib, Selumetinib, or Cobimetinib) or ERK1/2i (GDC-0994; Robarge et al., 2014) determined a delayed activation of ERK5 through a PDGFRi-sensitive pathway (Adam et al., 2020). Combined MEK5-ERK5 co-targeting using Trametinib + XMD8-92 or Trametinib + ERK5 genetic inhibition (shRNA) prevented long-term growth *in vitro*, thus supporting the relevance of ERK5 in the proliferation and survival of N-RAS-mutated melanoma cells upon MEK1/2-ERK1/2 inhibition. More importantly, Trametinib + XMD8-92 effectively repressed the growth of N-RAS-mutated melanoma xenografts. Therefore, these data demonstrated that MEK1/2i + ERK5i co-treatment could improve the effectiveness of available MEK1/2i therapies in N-RAS-mutated melanoma patients (Adam et al., 2020).

## Evidence for ERK5 Activation as a Resistance Mechanism in BRAF-Driven Cancers

Mutated BRAF is responsible for ERK1/2 pathway activation in above 50% of patients with advanced melanoma (Davies et al., 2002; Flaherty et al., 2012). Unfortunately, BRAFi monotherapy (i.e., using the BRAFV600Ei Vemurafenib) frequently fails as a consequence of a resistance mechanism which leads to ERK1/2 pathway reactivation (Hauschild et al., 2012; Shi et al., 2014; Van Allen et al., 2014). To overcome this resistance, combined inhibition of BRAF and MEK1/2 (CIBM) is among the current approaches used in melanoma patients harboring BRAF-activating mutations (Larkin et al., 2014; Long et al., 2014). However, resistance to CIBM can be also developed, and represents a major obstacle to the long-term clinical benefit of therapy (Samatar and Poulikakos, 2014). A recent report showed that ERK5 phosphorylation is enhanced in BRAF-mutated melanoma cells resistant to CIBM (Song et al., 2017). The demonstration that ERK5 activation is associated with this resistance was achieved showing that either genetic (shRNA) or pharmacological (XMD8-92) ERK5 inhibition impaired the acquisition of resistance to CIBM and sensitized resistant cancer cells to Vemurafenib and/or Trametinib, restoring the anti-proliferative effect of the latter. The activating phosphorylation of ERK5 in response to CIBM therapy seemed to be sustained by a SRC/MEK5 cascade. Consistently, either CIBM + XMD8-92 or CIBM + SRCi (Dasatinib) were more effective than CIBM alone in reducing the growth of BRAF-mutated melanoma xenografts, and showed the same effects as CIBM + XMD8-92 + Dasatinib. In the same paper, the authors proposed that BRAF could be responsible for SRC activation, thus positioning BRAF upstream of ERK5 in CIBM-resistant cells (Song et al., 2017). A later work further supported the key role of ERK5 in MAPKi resistance in BRAF-mutated melanoma (Benito-Jardón et al., 2019). Indeed, besides confirming the activation of ERK5 upon CIBM, it was shown that melanoma cells double-resistant to either Vemurafenib and Trametinib or to Vemurafenib and SCH772984 (Morris et al., 2013) displayed enhanced IGF-1R expression and kinase activity, as well as increased IGF-1R-dependent MEK5-ERK5 activation. Consistently, inhibition of IGF-1R with Linsitinib reduced the proliferation of SCH772984-resistant cells, and prevented the activation of ERK5 in CIBM- or Vemurafenib/SCH772984-resistant cells. In the latter, Linsitinib decreased the growth of spheroids in 3D cultures as well as in xenografts in NOD/SCIDgamma mice (Benito-Jardón et al., 2019). Finally, a recent work identified an additional mechanism linking ERK5 to MAPKi resistance in BRAFV600E-mutated melanoma cells (Lee et al., 2020). In the study, the authors showed that the treatment of BRAFV600E-mutated melanoma cell lines with Cobimetinib or Vemurafenib resulted in the increase of ERK5 phosphorylation, and demonstrated that this effect was mediated by Mir-211. In particular, the increased expression of Mir-211 upon Cobimetinib or Vemurafenib treatment was responsible for the inhibition of the expression of DUSP6, that resulted in ERK5 increased phosphorylation. Interestingly, DUSP6 overexpression prevented the increase in tumor growth occurring upon overexpression of Mir-211 in BRAFV600E-mutated melanoma xenografts. Consistent with a role for ERK5 in Mir-211 overexpressing cells, treatment with XMD8-92 or the MEK5 inhibitor BIX02189 reduced the proliferation of melanoma cells overexpressing Mir-211 (Lee et al., 2020). All above led to definitely include the ERK5 pathway among those involved in resistance to MAPKi in BRAFV600E-mutated melanoma cells.

## Evidence for ERK5 Activation as a Resistance Mechanism in ALK-Driven Cancers

The compensatory activation of ERK5 upon MEK1/2 targeting has also been reported in anaplastic lymphoma kinase (ALK)-addicted neuroblastoma cells (Umapathy et al., 2017). In this study, the authors found that the growth of N-RAS-mutated neuroblastoma cell lines and xenografts is sensitive to MEK1/2-targeting therapy, while that of ALK-addicted neuroblastoma cells and xenografts is not. Interestingly, ALK-addicted neuroblastoma cells treated with the MEK1/2i Trametinib showed an increased phosphorylation/activation of the AKT and ERK5 kinases, that the authors proposed to be responsible for a compensatory mechanism supporting cell proliferation. On the basis of a previous report from the same group, the activation of ERK5 in ALK-addicted neuroblastoma cells was proposed to be due to the PI3K-AKT-MEKK3-MEK5 axis (Umapathy et al., 2014). Overall, these studies suggest that ERK5 pathway inhibition in combination with MEKi might be regarded as a potential therapeutic strategy in ALK-addicted neuroblastoma (Umapathy et al., 2017).

## Demonstrated and Possible Mechanisms of ERK5 Activation Upon RAF-MEK1/2-ERK1/2 Targeting

**FIGURE 1:**
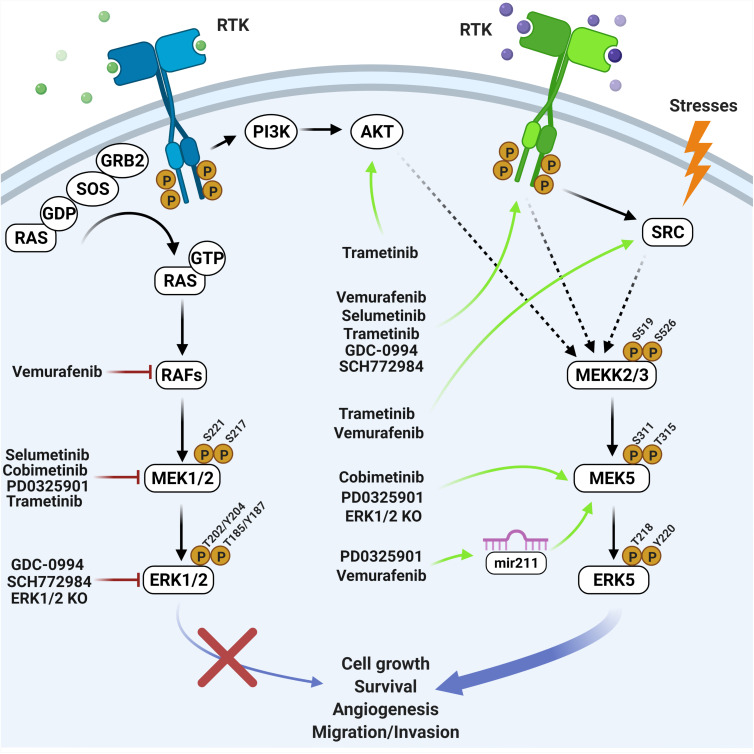
Overview of the effects elicited by RAF-MEK1/2-ERK1/2 inhibitors on the MEK5-ERK5 pathway. Black arrows indicate direct activation mechanisms. Dashed arrows indicate demonstrated but not direct mechanisms. Red lines indicate inhibiting treatments. Green arrows indicate resistance mechanisms occurring upon genetic or pharmacological inhibition of the ERK1/2 pathway (created with Biorender.com).

The above studies demonstrated the existence of a number of mechanisms responsible for MEK5-ERK5 activation following BRAF-MEK1/2-ERK1/2 inhibition (Figure 1). One of these mechanisms involved the increased expression of RTKs (Umapathy et al., 2017; Vaseva et al., 2018; Benito-Jardón et al., 2019; Adam et al., 2020). Additionally, as ERK1/2 activation may trigger a negative feedback directed to prevent an excessive level of activation of upstream activators, the pharmacological inhibition of ERK1/2 lead to loss of this feedback, resulting in a feedforward activation of RTKs (Lake et al., 2016) such as EGFR (Duncan et al., 2012; Lito et al., 2012). Both increased expression and activation of RTK resulted to be sufficient to activate MEK5-ERK5. Furthermore, the suppression of the above negative feedback elicited the activation of PI3K-AKT, leading to the subsequent increase of ERK5 signaling (Umapathy et al., 2014, 2017). Interestingly, even stronger evidence has been obtained that ERK5 activation itself leads to the activation of AKT (Lennartsson et al., 2010; Roberts et al., 2010; Bin et al., 2016), which in turn could strengthen the pro-survival role of ERK5 signaling in a context of resistance to treatment (Bera et al., 2014). Additional negative feedback mechanisms elicited by ERK1/2 involved DUSPs activation (Sarkozi et al., 2007). DUSPs prevented ERK5 phosphorylation, so that when MEK1/2-ERK1/2 is inhibited DUSPs inactivation resulted in enhanced ERK5 phosphorylation (de Jong et al., 2016). Along this line, DUSP6/MKP-3, initially reported to inactivate ERK1/2 but not ERK5 (Arkell et al., 2008), has been recently shown to participate in ERK5 activation following ERK1/2 pathway inhibition (Lee et al., 2020).

Besides the already elucidated mechanisms listed above, other compensatory processes underlying treatment resistance may occur upon RAF-MEK1/2-ERK1/2 targeting (Samatar and Poulikakos, 2014) and be mediated by upstream ERK5 activators (Stecca and Rovida, 2019). Among the latter, the MAP3K TPL2/COT (Chiariello et al., 2000) has been associated with *de novo* resistance to MEK1/2i or BRAFV600Ei in BRAF-mutated melanomas (Johannessen et al., 2010). Another possible mechanism may involve RAF-1, an additional possible ERK5 activator (English et al., 1999), the overexpression of which has been linked to acquired resistance to MAPKi (Samatar and Poulikakos, 2014). Additionally, BRAF, that may be amplified as a resistance mechanism to RAF-MEK1/2-ERK1/2 targeting (Samatar and Poulikakos, 2014), as well as BRAFV600E, has been recently demonstrated to activate ERK5 (Tusa et al., 2018). In support to the appropriateness of the dual targeting of the ERK5 and ERK1/2 pathways, in the same paper we showed that the combination Vemurafenib + XMD8-92 was more effective than either drug alone in reducing the growth of BRAF-mutated melanoma xenografts. Furthermore, Vemurafenib + XMD8-92 was necessary to reduce the amount of nuclear ERK5 (Tusa et al., 2018), which is critical for the support of cell proliferation (Raviv et al., 2004; Buschbeck and Ullrich, 2005; Iñesta-Vaquera et al., 2010; Gomez et al., 2016). Finally, CDK5, that plays a relevant role in tumorigenesis (Goodyear and Sharma, 2007; Eggers et al., 2011; Pozo et al., 2013), has been recently demonstrated to activate ERK5 (Zhuang et al., 2016). Because several reports have shown that CDK5 and ERK1/2 regulate each other, so that their activities may be inversely correlated (Sharma et al., 2002; Zheng et al., 2007; Banks et al., 2015), we may speculate that CDK5 may determine ERK5 activation upon ERK1/2 pathway inhibition.

## Concluding Remarks

Members of conventional MAPK pathways are among the most sought-after oncogenic effectors for the development of novel strategies to treat cancer (Kim and Choi, 2010; Braicu et al., 2019). Despite the fact that the MEK5-ERK5 pathway has been the less-studied of MAPK cascades, several lines of evidence pinpointed its relevance in cancer biology (Simões et al., 2016; Stecca and Rovida, 2019; Tubita et al., 2020). Furthermore, the literature summarized in this paper highlights the involvement of MEK5-ERK5 activation as a compensatory/resistance mechanism to RAF-MEK1/2-ERK1/2 targeting (Table 1). However, the mechanisms underlying the cross-talk between the ERK1/2 and the ERK5 pathways have not been fully elucidated, so that they should be further explored in the future in order to reinforce the rationale for a combined targeting of ERK1/2 and ERK5 pathways in order to achieve a more effective response in RAS-RAF-MEK1/2-ERK1/2-addicted cancer.

**TABLE 1:** Cancer specific ERK5-activating resistance mechanisms following RAF-MEK1/2-ERK1/2 targeting.

Cancer type	Genomic alteration supporting ERK1/2 pathway activation	Ineffective targeting (pharmacological/genetic inhibition)	ERK5-activating resistance mechanism	Effective combined targeting strategies	References
Colorectal cancer	K-RAS mutation	MEK1/2 (PD0325901) or ERK1/2 (KO)	Increased phosphorylation/activation of ERK5 likely due to DUSP deregulation	MEK1/2i + ERK5i (*in vitro*)	de Jong et al., 2016
Neuroblastoma	ALK mutation/amplification	MEK1/2 (Trametinib)	Increased activation of AKT-ERK5 signaling	MEK1/2i + ERK5i or AKTi (proposed)	Umapathy et al., 2017
Pancreatic ductal adenocarcinoma	K-RAS mutation	MEK1/2 (Selumetinib, Trametinib); ERK1/2 (SCH772984)	Upregulation of EGFR-SRC-ERK5 pathway	ERK1/2i + ERK5i (*in vivo*)	Vaseva et al., 2018
Non-small-cell lung carcinoma	K-RAS mutation	MEK1/2 (Cobimetinib)	Increased phosphorylation/activation of ERK5 likely dependent on RTKs	MEK1/2i + ERK5i or ERK5-KO (*in vitro*) or ERK5-KD (*in vitro* and *in vivo*)	Dompe et al., 2018
Melanoma	N-RAS mutation	MEK1/2 (Trametinib); ERK1/2 (GDC-0994)	Increased phosphorylation/activation of ERK5 likely dependent on PDGFRβ	ERK1/2i + ERK5i (*in vitro*); MEK1/2i + ERK5i (*in vitro* and *in vivo*)	Adam et al., 2020
Melanoma	BRAF mutation	BRAF + MEK1/2 (Vemurafenib + Trametinib)	Increased phosphorylation/activation of ERK5 mediated by SRC-MEK5 cascade	BRAFi/MEK1/2ì + ERK5-KD (*in vitro*) or ERK5i (*in vitro* and *in vivo*)	Song et al., 2017
Melanoma	BRAF mutation	BRAF + MEK1/2 (Vemurafenib + Trametinib); BRAF + ERK1/2 (Vemurafenib + SCH772984)	Upregulation of IGF1R-MEK5-ERK5 pathway	ERK1/2i + IGF1Ri (*in vivo*)	Benito-Jardón et al., 2019
Melanoma	BRAF mutation	BRAF (Vemurafenib); MEK1/2 (PD0325901)	Increased phosphorylation/activation of ERK5 mediated by miR-211		Lee et al., 2020

**KO, knock-out; KD, knock-down (shRNA).**

Many small-molecule compounds targeting ERK5 (including XMD8-92, XMD17-109, JWG-071, AX15836, BAY-885) or MEK5 (BIX02188, BIX02189) have been developed (Tatake et al., 2008; Yang et al., 2010; Deng et al., 2013; Lin et al., 2016; Wang et al., 2018; Nguyen et al., 2019) and exhibited remarkable effects in reducing the growth of human tumor xenografts in mice. Recently, an orally bioactive ERK5 inhibitor (Compound 46) was developed (Myers et al., 2016). However, it is worth point out that the off-target effects of XMD8-92 and derivatives (Deng et al., 2013; Wang et al., 2018) on BRD4 (Lin et al., 2016; Williams et al., 2016) certainly hampered the interpretation of the results obtained with these compounds, unless a genetic approach was provided to support the data obtained via drug treatment. On the other hand, some ERK5i (i.e., XMD17-109 and AX15836) cause a conformational change in the ERK5 kinase domain which leads to the exposure of the C-terminal NLS and to a paradoxical activation of the ERK5 TAD (Lochhead et al., 2020), enabling ERK5 to regulate its downstream targets. None of these inhibitors, however, has been tested in humans so far. TG02, a dual ERK5/CDK inhibitor, has been tested in clinical trials for hematological malignancies following the promising results obtained in preclinical studies (Alvarez-Fernandez et al., 2013; Ortiz-Ruiz et al., 2014). Based on all above, concerted efforts should be pursued to develop therapeutically suitable MEK5-ERK5 inhibitors. Indeed, besides representing a promising strategy for cancer treatment *per se*, ERK5 pathway inhibition should be exploited to prevent acquired resistance in cancers where inhibition of the RAS-RAF-MEK1/2-ERK1/2 cascade represents a valuable therapeutic option.

## Author Contributions

ER conceptualized this review. AT, IT, and ER wrote and revised the manuscript. All authors approved the final version of the manuscript.

## Conflict of Interest

The authors declare that the research was conducted in the absence of any commercial or financial relationships that could be construed as a potential conflict of interest.
